# Stem Cells as Drug-like Biologics for Mitochondrial Repair in Stroke

**DOI:** 10.3390/pharmaceutics12070615

**Published:** 2020-07-01

**Authors:** Jeffrey Farooq, You Jeong Park, Justin Cho, Madeline Saft, Nadia Sadanandan, Blaise Cozene, Cesar V. Borlongan

**Affiliations:** Department of Neurosurgery and Brain Repair, University of South Florida Morsani College of Medicine, Tampa, FL 33612, USA; jfarooq@usf.edu (J.F.); youjeongpark@usf.edu (Y.J.P.); justincho@usf.edu (J.C.); saftmad@umich.edu (M.S.); nas146@georgetown.edu (N.S.); bcozene@tulane.edu (B.C.)

**Keywords:** retinal ischemia, blood–brain barrier, endothelial, reactive oxygen species, oxidative stress, tunneling nanotubules, neuron, central nervous system, inflammation, hypoxia

## Abstract

Stroke is a devastating condition characterized by widespread cell death after disruption of blood flow to the brain. The poor regenerative capacity of neural cells limits substantial recovery and prolongs disruptive sequelae. Current therapeutic options are limited and do not adequately address the underlying mitochondrial dysfunction caused by the stroke. These same mitochondrial impairments that result from acute cerebral ischemia are also present in retinal ischemia. In both cases, sufficient mitochondrial activity is necessary for cell survival, and while astrocytes are able to transfer mitochondria to damaged tissues to rescue them, they do not have the capacity to completely repair damaged tissues. Therefore, it is essential to investigate this mitochondrial transfer pathway as a target of future therapeutic strategies. In this review, we examine the current literature pertinent to mitochondrial repair in stroke, with an emphasis on stem cells as a source of healthy mitochondria. Stem cells are a compelling cell type to study in this context, as their ability to mitigate stroke-induced damage through non-mitochondrial mechanisms is well established. Thus, we will focus on the latest preclinical research relevant to mitochondria-based mechanisms in the treatment of cerebral and retinal ischemia and consider which stem cells are ideally suited for this purpose.

## 1. Stroke: A Trilogy of Cell Death Events

Stroke is currently the fifth leading cause of death in the United States and can cause disabling neurological deficits including cognitive impairment, hemiparesis, sensory disturbance, and aphasia [1]. Studies project that by 2030, 3.88% of the US population over the age of 18 will have a stroke and the total annual stroke-related costs will reach $240.67 billion [1]. Despite an emphasis on implementing effective acute and chronic stroke care made by the American Heart Association and American Stroke Association, there are only two FDA-approved treatment options available for acute stroke: tissue plasminogen activator (tPA) and endovascular thrombectomy. Unfortunately, their use is limited by the short therapeutic time window and risk for additional damage. Although rehabilitation is an option for chronic stroke care, functional recovery remains modest. Ischemic stroke comprises 87% of all stroke cases and involves inadequate blood perfusion to vital organs like the brain, which leads to oxygen and nutrient deprivation and subsequent cell death [2,3]. With the central nervous system’s limited capability to recover after an injury, a treatment strategy to restore neurological function is an unmet need.

**Figure 1:**
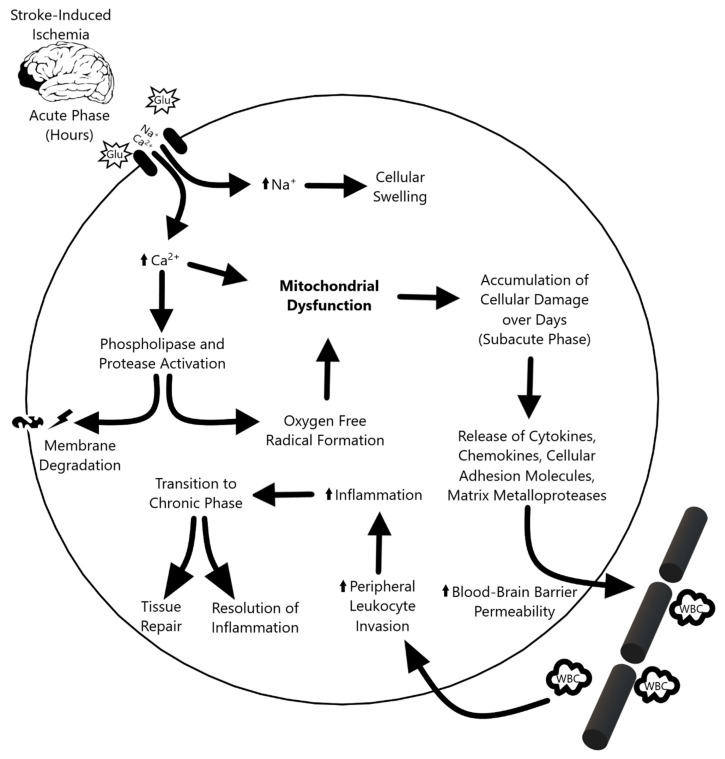
Overview of the three phases of the ischemic cascade. The acute phase following stroke-induced damaged precipitates ionic imbalances that ultimately propagate mitochondrial dysfunction. This progresses to subacute phase-based cellular damage, leukocyte invasion, and inflammation. Inflammation dissipates in the chronic phase, which culminates in tissue repair.

The ischemic cascade (Figure 1) triggered by stroke can be divided into three phases. During the acute phase—within the first few hours after stroke—blood flow, ATP, and energy stores in the tissue plummet, causing ionic disruption, mitochondrial dysfunction, and metabolic failure. The ionic imbalance and release of neurotransmitters spike the influx of sodium and calcium into the cell. This increased intracellular calcium activates phospholipases and proteases that degrade integral proteins, while the surplus of sodium leads to cellular swelling. [4] Furthermore, an increase in oxygen free radicals and other reactive oxygen species causes further damage and cell death during the acute phase [5,6]. The subacute phase follows, which lasts for the first few days after the ischemic event. Injured cerebral tissue releases cytokines, chemokines, cellular adhesion molecules, and matrix metalloproteases (MMPs), which increases the permeability of the blood–brain barrier (BBB) and attracts peripheral leukocytes to infiltrate and upregulate inflammation [4,7]. In the transition to the chronic phase, the inflammation resolves and tissue repair begins, but the endogenous repair process is not sufficient to confer functional recovery in stroke patients. Although the mechanism is not yet fully understood, the chronic phase is marked by the re-establishment of homeostasis and suppression of the inflammatory response. Like the neurological and cognitive deficit associated with cerebral stroke, retinal ischemia is characterized by visual impairment caused by lack of blood flow to the eye which results in a cascade of apoptotic events, oxidative stress, and mitochondria dysfunction in retinal ganglion cells [8]. Retinal ischemia is one of the major contributors to visual impairments caused by stroke and research suggests that mitochondria play a critical role in determining ganglion cell survival [9,10]. With the overlapping pathologic characteristics between cerebral and retinal ischemia in mind, the role of mitochondrial dysfunction in stroke offers a unique window to not only further the understanding of stroke pathology, but also to develop treatment strategies.

## 2. May the Force of Mitochondria Be with Stroke

Mitochondrial dysfunction plays a key role in the pathological progression of ischemic stroke [11]. Mitochondria are ubiquitous, double membrane-bound organelles that contain electron transport proteins, the ATP synthetase complex, and ATP/ADP transport proteins. They generate energy in the form of ATP by coupling the transfer of electrons from FADH2 and NADH through the electron transport chain with the phosphorylation of ADP. The majority of ATP generated in cerebral tissue is funneled into neuronal electrogenic activity [12]. Therefore, sufficient energy supply by the mitochondria is critical for neuronal excitability and survival. The mitochondria also produce reactive oxygen species (ROS) and play a role in regulating apoptosis—functions tightly linked to the pathology of ischemic stroke [11]. As in the case of many neurological disorders like amyotrophic lateral sclerosis and Parkinson’s disease, cerebral ischemia leads to ROS overproduction, which compromises the functional and structural integrity of brain tissue [13,14]. During ischemic stroke, insufficient blood circulation deprives cerebral tissue of glucose and oxygen. This disruption prevents the mitochondria from performing oxidative phosphorylation, a process that generates 92% of the overall cellular ATP, and cells are no longer able to maintain their metabolic functions [11,15,16]. Furthermore, the defective oxidative metabolism leads to excess production of ROS and reactive nitrogen species (RNS) following the re-establishment of blood flow during reperfusion [11]. The oxidative stress overwhelms the neutralization capacity of the endogenous antioxidant system, and the overproduction of ROS and RNS ultimately results in cell death through the destruction of proteins, lipids, and DNA [11].

The body has multiple mechanisms to combat oxidative stress and clear damaged mitochondria. Cells can sequester functional mitochondria while degrading dysfunctional mitochondria via mitophagy [11,16]. In addition, increased permeability of damaged mitochondrial membranes allows the release of pro-apoptotic molecules into the cytoplasm and signals apoptotic cell death [11]. At the molecular level, creatine kinase (CK) enzymes transfer the phosphate from creatine phosphate to ADP, generating ATP and creatine. Cytosolic CK enzymes act as an intracellular energy buffer and expression of mitochondrial CK is upregulated during times of energy deprivation [16,17,18]. However, in the setting of ischemic stroke, the damage and oxidative stress are often beyond the scope of repair by endogenous mechanisms. For example, CK enzymes are also susceptible to damage by ROS and RNS, and post-stroke upregulation can lead to a buildup of crystalline mitochondrial CK inclusion bodies that exacerbate mitochondrial dysfunction [16,17,19,20]. Taken together, the role dysfunctional mitochondria play in the ischemic cascade presents opportunities to develop therapies for acute and chronic stroke. This review will cover the latest preclinical data that support stem cell-based approaches to repair dysfunctional mitochondria and restore/protect neurological function.

## 3. The Return of the Force: Enhancing Mitochondrial Function in Stroke

The discovery that stem cells can replace dysfunctional mitochondria in damaged cells has primed the field of stroke therapy for critical improvements in treatment outcomes [21]. In the presence of pathological stress, a series of cellular signals induce the formation of molecular bridges between stem cells and damaged cells through which mitochondria can travel [22]. Microvesicles, nanotubules, and gap junctions often form these intercellular connections, although transfer can also occur through direct uptake or cell fusion [23]. Regardless of the method of transit, stem cell-mediated mitochondrial replacement results in the restoration of cell function [24].

The ideal cell source to leverage the therapeutic benefits of mitochondrial transfer is bone marrow-derived mesenchymal stem cells (BM-MSCs). This cell type gives rise to endothelial progenitor cells (EPCs), which produce nearly all of the endothelial cells in the body [25]. In the context of stroke, EPCs repair damaged tissue and improve long-term outcomes by migrating through the blood–brain barrier (BBB) to the site of injury to promote angiogenesis [26,27]. This EPC-induced vascular regeneration is possibly due to mitochondrial transfer; however, there are other potential mechanisms through which the repair may occur, such as the release of pro-angiogenic factors from the EPCs themselves. To confirm the mechanism of repair is EPC-mediated mitochondrial transfer, three conditions must be true: EPCs must be able to export mitochondria, endothelial cells must take up those mitochondria, and the uptaken mitochondria must be able to restore endothelial cell function.

An in vitro stroke model proved that EPCs could successfully discharge their mitochondria [27]. Protein analysis of EPCs—identified via the cellular markers CD34, Flk-1, lectin-UEA, and vWF—showed enhanced expression of the mitochondrial membrane protein TOM40 and increased ATP concentration. The increased level of these two cellular components is an indication that mitochondrial production is upregulated in the post-stroke environment [27]. Additionally, the finding of mitochondria that exist within extracellular vesicles derived from EPCs demonstrates the presence of a mitochondrial export mechanism [27]. Furthermore, based on their oxygen consumption rates, the extracellular mitochondria function at a standard capacity [27]. Finally, the levels of extracellular mitochondria produced by EPC-derived cultures were similar to that of other cell types [27]. Taken together, this is strong evidence that EPCs can release sufficient amounts of active, viable mitochondria.

The next step in confirming a mechanism of EPC-mediated mitochondria transfer is to assess whether endothelial cells take up these extracellular mitochondria and whether this would aid the recipient cell. In that same study, confocal microscopy demonstrated evidence of mitochondria-containing, EPC-derived extracellular vesicles within endothelial cells in the cerebral vasculature [27]. Therefore, EPCs can release mitochondria, and brain endothelial cells can uptake them. There is also evidence that upon absorption of the EPC-derived mitochondria, capillary-like appendages form on the endothelial cells and assist in angiogenesis [27]. Another interesting observation in brain endothelial cells exposed to EPC-conditioned media is reduced membrane permeability and upregulated VE-cadherin production, a cytoskeletal stabilization protein that promotes cell-cell adhesions [27,28]. Exposure of these same endothelial cells to oxygen and glucose deprivation (OGD) conditions rescued intracellular mitochondrial DNA levels, supporting a mechanism where EPC-mediated mitochondrial transfer not only provides immediate benefit to the recipient cell via healthy donor mitochondria but also restores the ability of the recipient cell to produce mitochondria [27].

Although at this point there is considerable evidence for the role of EPCs in restoring mitochondrial function via a transfer mechanism, there was still much uncertainty surrounding the benefits of stem cell mitochondrial therapy. Additional studies on EPC-derived mitochondria addressed these concerns. FACS-assisted proteome analysis of OGD-exposed brain endothelial cells reveals that uptake of EPC-derived mitochondria enhances the production of angiogenic and BBB proteins, including Serpin E1, plasminogen, FGF-4, and bFGF [27]. These findings illustrate the tremendous therapeutic potential of EPCs in post-stroke recovery by improving mitochondrial function, restoring BBB function, and promoting angiogenesis.

The ischemic conditions brought about by stroke damage endothelial cells and predispose them towards undergoing intrinsic pathway apoptosis, which is mediated by mitochondrial dysfunction [29]. Integrating a stem cell-based mitochondria treatment ameliorates this issue. However, it does not further our understanding as to why the transfer of mitochondria provides neuroprotective effects against ischemia of neurons. Elucidating this mechanism will significantly advance the current understanding of stem cell mitochondrial therapy. Immunofluorescent imaging and the Seahorse or Clark electrode assays will be useful in evaluating the functionality of mitochondria transferred into neurons [16]. These techniques facilitate visual inspection of the mitochondria and quantification of the bioenergetic recovery associated with their intercellular transfer.

Importantly, astrocytes can also transfer their mitochondria to damaged neurons but do so in a transient fashion. Thus, they do not produce the same neuroprotective effects that occur via stem cell transplantation and cannot prevent secondary cell death [30]. Given the incredible therapeutic promise of stem cell treatment for stroke, it is essential to understand the mechanistic difference between these two approaches and why only stem cells confer neuroprotection.

Examination of the electron transport chain (ETC), specifically complexes I–IV, can be performed using ETC complex inhibitors to study each portion of the chain in isolation. Mouse models with mutated mitochondria are instrumental in this investigation in order to definitively determine whether it is the mitochondria themselves that confer the neuroprotection or whether a different characteristic of the stem cell is responsible [16]. When directly comparing EPC-cultured models to Rho0 (dysfunctional mitochondria) models, the results were similar. In both scenarios, graft survival was less than 1% in the first two weeks and was too low to measure between weeks four through twelve [27]. As neuroprotection is more vital than graft survival in EPC-mediated mitochondrial transfer, this corroborates the hypothesis that the Rho0-cultured cells did not gain any neuroprotective benefit.

The safety of BM-MSCs compared to other sources of stem cells is relatively well established [31]. Nevertheless, it is always valuable to deliberate on the safety profile compared to the therapeutic potential. There are three main concerns when discussing the use of EPCs as a stem cell transplant source. First, EPCs enhance vessel formation; therefore, the tumorigenicity is of particular concern in patients who have pre-existing tumors [32]. Second, the ability of EPCs to promote angiogenesis via endothelial growth factor signaling can cause cerebral edema [33]. Third, EPCs may encourage cerebral inflammation by recruiting monocytes and releasing interleukin-8, a proinflammatory cytokine, although this is controversial and is challenged by recent research that demonstrates the opposite phenomenon of inflammatory modulation [34,35]. Therefore, with mild exceptions, the use of EPCs appears to be a safe and promising avenue for stroke therapy.

## 4. Force in the Outer Rim: Stroke Extends to the Retina but may be Repaired by Stem Cell-Mediated Mitochondria Transfer

Up to this point, we concentrated on literature that characterizes the transfer of mitochondria from stem cells to endothelial cells and neurons in the context of the ischemic brain. However, stroke patients often suffer maladies that extend beyond the brain, and complete functional improvement must address these aspects of recovery as well. A prime example of this is that ischemic stroke may cause damage to the eye, leading to visual impairment and a significant delay in recovery [36,37]. Importantly, the same pathology-associated changes in mitochondrial activity that occur in the case of ischemic stroke also underlie cell survival and death in retinal ischemia [9,10].

It is valuable to understand the role of mitochondrial dysfunction in cerebral and ocular disease post-stroke due to the markedly similar pathology and treatment options between these two conditions [38]. In addition, given the benefits of MSC therapy in restoring mitochondrial function, it is plausible to infer that MSC treatment will have analogous effects in retinal ischemia, potentially diminishing the ischemia-induced cell death [38]. The use of a middle cerebral artery occlusion (MCAO) rat model and retinal pigment epithelium (RPE) cell culture model of OGD are beneficial for studying retinal ischemia, as they accurately reproduce the same symptomology in vivo and in vitro, respectively [38]. Following MCAO, blood perfusion to the ipsilateral hemisphere of the brain and ipsilateral eye decreases by 80%, a remarkably similar decrease to that seen in retinal and cerebral ischemia [38,39,40]. Upon resolution of the ischemia, blood flow returns to the ipsilateral eye and hemisphere nearly five minutes faster than the contralateral side due to angiogenesis and neovascularization of the affected tissues [38,41,42]. However, the perfusion rate stabilizes between the two eyes within three days of stroke, owing to the lack of collateral circulation [43,44,45]. On days 3 and 14 following MCAO, immunohistochemical analysis reveals insufficient blood flow to the retina, corresponding to a decrease in retinal ganglion cell survival and an increase in the rate of degeneration of the optic nerve [38]. Hemispheric blood flow post-stroke was also reduced [38]. RPE cell death is also increased in the in vitro OGD model [38]. Importantly, ischemic insult and the severe loss of healthy retinal cells coincides with mitochondrial dysfunction in both in vivo and in vitro models. Therefore, inducing ultrastructural defects in mitochondria is an effective model to study the pathological course of retinal ischemia [38].

There are considerable benefits to using MSC therapy to treat ischemia-induced eye damage, such as enhanced preservation of retinal ganglion cells. The decreased cell death is likely due to improved mitochondrial function, which may be due to MSC-derived mitochondrial transfer to the retinal ganglion cells [38]. MSC transplantation rescues the function of the electron transport chain within mitochondria, which also restores the energy balance of the cell. In addition, it ameliorates ganglion cell loss and optic nerve damage at the 14 days post-treatment [38]. RPE cells co-cultured with MSCs demonstrate improved survivability following OGD likely due to the restored network morphology, dynamics, and respiratory capacity of the mitochondria [38]. Sheltering mitochondrial DNA, improving respiration, and promoting mitochondrial signaling, the structure of mitochondrial networks is likely regulated by the balanced interaction between fission and fusion of the mitochondria [38]. However, when mitochondrial homeostasis is not present, typically when fission surpasses fusion, the mitochondria breaks apart into isolated, rounded mitochondria fragments [38]. The dynamics and configuration of the mitochondria and its network in vitro show that RPE cells co-cultured with MSCs possess much larger networks with less isolated, rounded mitochondrial fragments [38]. Additionally, stem cells that co-culture with MSCs reproduce standard expressive levels of the fusion protein mitofusin-2, which is downregulated by OGD insult [38,46,47]. MSC transplantation, however, does not increase the activity of OGD-induced fission protein dynamin-related protein-1 [39,48]. This is the first study to report about the ability of MSCs to regulate depolarization of the mitochondrial membrane by OGD [38].

The interaction of creatine and phosphocreatine conversion prevents lapses in cellular energy supplies under healthy conditions. However, it is still possible for the energy supply to be damaged under ischemic conditions, worsening mitochondrial dysfunction. Creatine supplementation possesses therapeutic characteristics in various neurodegenerative disorders that may address the lack of energy supply and the normal function of the mitochondria [49]. Therefore, creatine supplementation can rescue endangered mitochondria along with a host of other wide-reaching positive effects. Furthermore, stroke models that reveal the therapeutic benefits of creatine treatment also support this notion [50,51]. These non-clinical experiments highlight mitochondrial functioning as a key player for cerebral and retinal ischemia pathology. Consequently, the transfer of healthy mitochondria by stem cells moderates the restoration of mitochondrial function. By recovering the mitochondria, stem cells offer a potential treatment to restore the morphology and function of damaged neural and optic cells.

Based on previously mentioned studies, transferring healthy mitochondria by exogenous MSCs can potentially restore respiratory functions in ischemic retinal cells, reducing cell loss. Future studies should investigate how MSCs successfully transfer healthy mitochondria to retinal ganglion cells. Studies should also observe and describe the metabolic and proteomic properties of MSC-derived mitochondria post-transplantation into ischemic retinal cells. EPCs’ affinity for BBB repair makes them a favorable MSC subtype. EPCs are known for being safe and effective to use in stem cell therapy. Additionally, their ability to donate healthy mitochondria justifies the need to further investigate its effects on retinal ischemia. However, the study presents a significant limitation. Specifically, no known study provides a detailed report regarding the physical characteristics of MSC that are involved in the mitochondrial transfer in retinal ischemia [38]. The function and phenotypic properties of EPC-derived mitochondria in cerebral stroke are explained in detail. However, the claim that EPCs are a key player in mitochondrial transfer in retinal ischemia lacks sufficient evidence. Future studies should focus on this distinct function of MSCs and EPCs to interconnect this gap in knowledge on mitochondria-mediated regenerative medicine of ischemic diseases.

## 5. The Rise of the Force: New Horizons in Mitochondrial Repair for Stroke

Stroke-induced ischemia results in insufficient oxygen delivery to cells and prevents mitochondria from performing cellular respiration. The ensuing loss of ATP is not compatible with cellular viability and ultimately precipitates mitochondrial dysfunction and cell death. In response to stroke and stroke-like lesions, cells upregulate mitochondrial synthesis to compensate for damaged mitochondria [52]. A variety of techniques can measure this new production of mitochondria, as well as the functional capacity of existing mitochondria. Therefore, mitochondria are potent biomarkers for stroke and ischemic brain injury. In addition, mitochondria are crucial organelles to target for therapeutic strategies due to their neuroprotective potential that helps bolster post-stroke recovery.

Despite the innovations in stroke therapy over the past decade, novel methods of research continue to elucidate fundamental information on the mechanistic role of mitochondria in stroke and enable the development of powerful new therapeutic strategies. A novel technology, Seahorse XFe24, measures mitochondrial respiration and allows investigators to directly detect changes in cellular energetics, rather than relying on cellular signaling [53]. This technique has the additional advantage of requiring only a small sample of mitochondria from each region of the brain for accurate analysis. Thus, Seahorse XFe24 is a convenient method to extract mechanistic data about mitochondrial function from experiments on mitochondrial repair in stroke. Along with novel technologies, several new strategies designed to target the mitochondria as therapeutics have emerged and are discussed below (Table 1).

### 5.1. Pharmacological Treatment

Several pharmaceutical agents, such as the dopamine D2 receptor antagonist pramipexole (PPX), facilitate neuroprotection through the action of mitochondria. Administration of PPX to transient MCAO model rats after an ischemic stroke reduces infarct volume, neurological deficit severity, mitochondrial ROS formation, mitochondrial calcium concentration, and swelling of the mitochondrial membrane, while simultaneously increasing oxygen consumption and the respiratory control ratio [54]. Therefore, PPX is a promising treatment for ischemic stroke due to its ability to inhibit mitochondria-mediated cell death and improve neurological functions and motor strength.

The pharmacological administration of tetrahydrocurcumin (THC) also alleviates mitochondrial dysfunction and improves functional capacity and motor coordination. THC epigenetically reduces plasma and tissue homocysteine (Hcy) levels and Hcy-induced mitochondrial oxidative stress [55]. THC treatment of MCAO model mice compared to control mice significantly improves neuroscores, strengthens coordination and neuromuscular function, reduces cerebral blood flow, prevents damage from increased permeability in the brain interstitial parenchyma, decreases cerebrovascular permeability, modulates Hcy levels, and significantly reduces matrix metalloproteinase-9 levels [55]. Thus, THC’s ability to alleviate oxidative stress and reverse ischemia-induced changes in mitochondria gives it potential as a treatment for ischemic stroke.

Another drug-based approach to mitochondrial dysfunction is the use of nicotinamide mononucleotide (NMN) to increase NAD+ levels. NMN extends the lifespan of mice with the mitochondrial disease Leigh Syndrome by normalizing NAD+ redox imbalance and lowering H1F1a accumulation in skeletal muscle [56]. Furthermore, NMN elevates alpha-ketoglutarate (KG) production and suppresses hypoxic signaling [56]. Direct administration of a cell-permeable form of KG also extends lifespan and delays the onset of neurological phenotype [56]. The encouraging results of NMN and KG treatment in Leigh Syndrome warrants their consideration for mitochondrial damage from stroke.

Cationic arginine-rich peptides (CARPs) are another candidate pharmacological therapeutic that specifically enhance mitochondria-mediated neuroprotection in stroke. CARPs are small peptides, composed of up to 30 amino acids, that cross the BBB and localize to mitochondria in neurons. This property alone highlights their value as a pharmacologic treatment for stroke, as many other promising drugs do not effectively cross the BBB, making their administration difficult or ineffective. Once in the mitochondria, CARPs efficiently eliminate ROS that accumulate during ischemia and restore proper function of the mitochondria, rescuing the cell from free radical damage and reestablishing cellular viability [57].

### 5.2. Autophagy/Mitophagy

Although restoring the respiratory functions of mitochondria improves stroke outcomes, this is not the only strategy for recovery. Autophagy, the process of degrading and recycling damaged or unnecessary cellular components, can also mitigate mitochondrial dysfunction. A high salt diet is particularly dangerous because it increases the risk of hypertension-related stroke occurrence, reduces the efficiency of autophagy, and downregulates the production of the electron transport chain enzyme NDUFC2 [58]. However, the pharmacologic agent Tat-Beclin 1 restores autophagy activity, thereby mitigating stroke occurrences. This underscores the critical role of autophagy in normal mitochondrial function and suggests a possible pharmaceutical approach to treat hypertension-related stroke.

The proteins ULK1, NDP52, and TANK-Binding Kinase 1 (TBK1) are also meaningful targets for stroke therapies through targeting autophagy. TANK1 recruits the ULK1 complex to the NDP52 receptor to initiate autophagy under conditions of starvation [59]. Therefore, upregulating NDP52 on mitochondria enhances autophagy and can improve stroke outcomes by promoting the selective recycling of damaged mitochondria.

Another potential target of autophagy-based treatments involves the small molecule Compound R6 and its regulation of mitochondria-mediated apoptosis. The release of cytochrome c from damaged mitochondria activates the intrinsic apoptotic caspase-9/3 cascade and results in cell death [60]. Compound R6 represses apoptosis and activates autophagy by blocking cytochrome c release and restricting mammalian target of rapamycin (mTOR) activity, respectively [60]. It is also potentially a neuroprotective agent as it crosses the blood–brain barrier and accumulates in the brain after intravenous injection [60]. The capability of Compound R6 to improve retinal and cerebral cell survival post-stroke by inhibiting apoptosis and triggering autophagy of damaged mitochondria warrants its further study.

Along with autophagy, manipulating the discrete molecular mechanisms of mitochondria to enhance mitophagy is another approach to ameliorate stroke-induced mitochondrial dysfunction. Cell cycle progression in the presence of impaired mitochondria generates damaged daughter cells, further exacerbating tissue injury and delaying recovery. However, the serine/threonine kinase PINK1 and the E3 ubiquitin ligase Parkin mediate the elimination of these impaired mitochondria through induction of TBK1 to upregulate mitophagy, the targeted recycling of mitochondria. By directing TBK1 to the mitochondrial membrane, away from its role at the centromere during mitosis, PINK1 and Parkin halt the cell cycle at the G2/M phase [61]. Therefore, PINK1 and Parkin activation may mitigate ischemic injury and improve stroke outcomes.

In addition to enhancing TBK1-mediated mitophagy, PINK1 upregulates the Parkin-induced mitophagy pathway in the presence of damaged mitochondria. During an ischemic injury, the depleted mitochondrial membrane potential inhibits PINK1 importation to the inner mitochondrial membrane (IMM). Instead, PINK1 binds to Tom 7, accumulates on the outer mitochondrial membrane (OMM), then activates Parkin-induced mitophagy. However, the IMM-resident protease OMA1 cleaves PINK1 in the absence of Tom 7, abolishing mitophagy [62]. Hence, suppression of OMA1 promotes mitophagy and may act as a therapeutic tool in alleviating stroke-induced mitochondrial damage.

While the PINK1/Parkin pathway is valuable to preserve non-neuronal cells, Parkin-induced mitophagy is not as effective in neurons as only a small fraction of mitochondria in axons undergo mitophagy [63]. However, the Mul1/Mfn2 pathway is a valuable target to protect neuronal mitochondrial integrity under long-term stress. Mfn2, which normally mediates mitochondrial fusion and interaction with the endoplasmic reticulum (ER), is enhanced in the absence of Mul1, leading to hyperfusion and blockage of ER-Mito interactions. The loss of ER–mitochondria contact indirectly stimulates mitophagy. For this reason, regulating the Mul2–Mfn2 pathway, either by depleting Mul1 or overexpressing Mfn2, may be a useful therapeutic tool for stroke-induced mitochondrial damage.

### 5.3. Molecular and Other Mechanisms

Multiple cellular pathways spur mitochondrial deterioration in ischemic stroke. Apoptosis in mitochondria is upregulated during ischemic stroke and is associated with continuous mitochondrial permeability transition pore (MPTP) opening in the inner and outer mitochondrial membranes [64]. ROS production in the mitochondrial respiratory chain causes tissue damage during reperfusion. When oxygen returns to hypoxic brain tissues, it generates superoxide free radicals, which then cause oxidative damage and calcium accumulation. This leads to MPTP induction during ischemia that causes a decrease in mitochondrial membrane potential, depolarization of the mitochondria, swelling of the mitochondrial substances to the cytoplasm, and ultimately organ dysfunction and cell death [65,66]. Therefore, the key to protecting the mitochondria from oxidative damage is by using exogenous antioxidants during ischemic reperfusion (IR) injury and inhibiting MPTP induction. Specifically, using mitochondrial-targeted antioxidants protects against stroke-induced damage [67].

Mitochondrial transporters are also important in preventing atherosclerosis, the accumulation of fatty plaques on the inner wall of arteries, which is a significant risk factor for stroke. Poor metabolism or excess intake of lipids intensifies atherosclerotic plaque buildup and heightens the chance of stroke and ischemic brain injury. The mitochondrial calcium uniporter (MCU) prevents lipid accumulation and maintains appropriate bioenergetics by facilitating oxidative phosphorylation in the mitochondria [68]. However, the loss of MCU activity results in poor oxidative phosphorylation and the accretion of lipids on the arterial wall, thus promoting atherosclerosis and stroke [68]. Therefore, maintaining or enhancing the function of the MCU is another viable mitochondria-based strategy for anti-stroke therapeutics.

Another source of mitochondrial dysfunction that may exacerbate stroke pathology is the mitochondrial ADP/ATP carrier. Mitochondrial ADP/ATP carriers are located in the impermeable mitochondrial membrane and transport ADP into the mitochondrial matrix and ATP out for use as energy [69]. Inappropriate cellular energetics caused by ischemia may hinder the activity of the ADP/ATP carriers. Developing novel techniques to target these transporters may help maintain adequate ADP/ATP transport and improve stroke outcomes.

Astrocytes transfer their healthy mitochondria to neurons in the peri-infarct area post-stroke, and this endogenous neuroprotective mechanism is a candidate for stroke-therapy. In particular, the nuclear and desmosome-associated protein Pinin (Pnn) upregulates anti-apoptotic Bcl-2 expression, promotes ERK signaling, reduces pro-apoptotic cleaved caspase-3 production, and enhances astrocyte survival. Therefore, therapeutically augmenting Pnn expression may improve the endogenous capacity of astrocytes to protect neurons and repair ischemic tissue in the brain [70].

**Figure 2:**
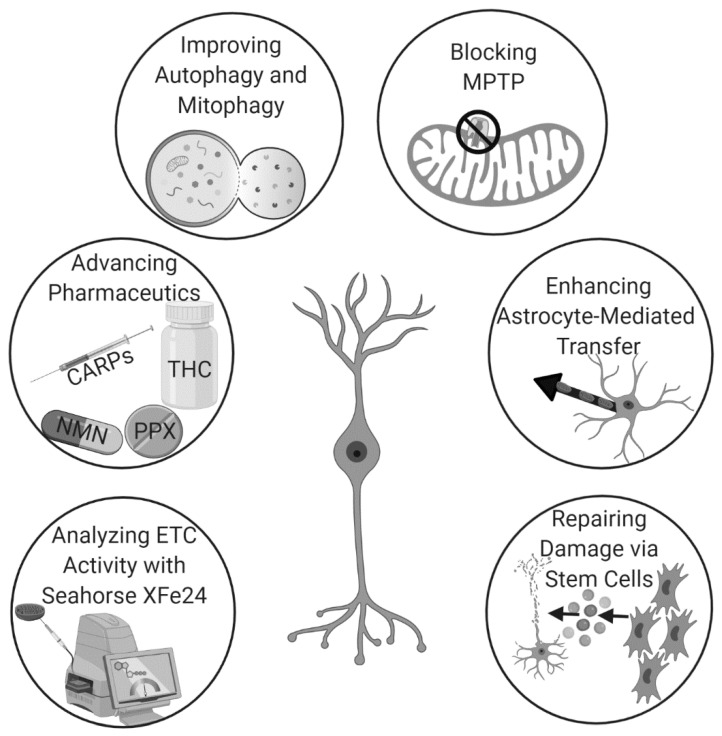
The use of mitochondrial repair for stroke is rapidly gaining momentum due to recent advancements in analytics, pharmaceutics, techniques that augment endogenous mechanisms, and stem cell therapy.

Hyperbaric oxygen therapy (HBOT), which delivers pure oxygen to patients in special high-pressure rooms, modulates inflammation in traumatic brain injury (TBI) when given after the onset of tissue damage. However, pretreatment with HBOT also reduces cell death and improves post-stroke outcomes by inducing endogenous astrocyte-based mitochondrial transfer to neuronal cells. The prophylactic use of HBOT to enhance neuroprotection circumvents the need for invasive surgical treatment and potentially toxic drug-based approaches [71]. Additionally, many hospitals already own HBOT chambers due to their therapeutic benefits in the treatment of various other ailments. Thus, there is already a strong framework for the use of HBOT-based mitochondrial transfer as a pretreatment for stroke to improve outcomes. Thus, there exists many unique and potentially efficacious pathways to augment mitochondrial repair for stroke therapy (Figure 2).

### 5.4. Stem Cells

Mitochondrial repair for stroke highlights the integral role of mitochondrial function on cell survival and neurological improvement. Mitochondrial dysfunction closely accompanies post-stroke secondary cell death. The hypoxic environment reduces energy production, further exacerbating stroke symptoms. Identifying a reliable method of mitochondrial transfer represents the first step towards developing an effective stroke treatment and is crucial to restoring cell function [72]. To this end, the transfer of healthy mitochondria from stem cells to areas of infarction stands as a potential avenue of therapy (Table 2). Stem cells transfer mitochondria via intercellular mechanisms to restore mitochondrial function in damaged cells, amplify cellular survival signals, and reprogram differentiated cells. Furthermore, they also mediate mitochondrial transfer from astrocytes into damaged neurons, enhancing neuronal vitality. Mitochondrial transfer thus serves as a viable means to reduce the chance of stroke-induced neuronal death and to restore the function of damaged cells [66].

While the benefits of stem cell therapy are clear, the mechanism is not. Determining the precise location of transfer between the donor and recipient cells will provide crucial insights into the mechanism and therapeutic actions of mitochondria. Such knowledge will guide efforts to optimize transfer conditions and maximize the beneficial effects of stem cells [73]. To this end, current research focuses on understanding the mechanism underlying mitochondrial transfer from stem cells into the stroke impacted brain. Cells utilize tunneling nanotubules (TNTs) or extracellular vesicles to transport mitochondria and other organelles between one another. Previous research has already shown that cell-to-cell signaling is involved in guiding healthy mitochondria from stem cells into damaged cells to rescue cell function. Intracellular quality of control helps maintain the functions of the mitochondria and combines fusion, fission, and degradation. For example, fusion of healthy and damaged mitochondria enables the exchange of DNA, proteins, and metabolites to prevent the buildup of damaged contents [66].

Although mitochondrial transfer is theoretically bidirectional, molecular signals released by damaged cells preferentially direct the movement of mitochondria and mtDNA from MSCs to damaged neurons [74]. One such signal is the activation of the pro-apoptotic protein caspase-3, which induces healthy mitochondria to move from MSCs to PC12 cells [64]. In addition, impaired retinal ganglion cells secrete proinflammatory cytokines, such as tumor necrosis factor-alpha and NF-kB, that initiate mitochondrial transfer from iPSC-MSCs [64]. Furthermore, extracellular vesicles that contain mitochondria released from the astrocytes help rescue neurons from ischemic stroke conditions and trigger CD38 to upregulate the release of mitochondria [66].

Deleting mitochondria from stem cells abolishes the regenerative benefits they confer to damaged endothelial cells, supporting the hypothesis that these organelles are responsible for repair. Furthermore, transferring mitochondria directly into ischemic brain tissue restores cellular energetics, facilitates homeostasis of the central nervous system (CNS), and resolves the inflammation that causes secondary cell death [38]. Stem cells afford vital protection to neurons and mitochondria in ischemic stroke, as well as in facilitating neuroprotection [75]. The following types of stem cells display a robust capacity to transfer healthy mitochondria to damaged neurons: Wharton’s jelly MSCs, iPSCs, BM-MSCs, EPCs, and adipose-tissue-derived stem cells [64].

MSCs are the most common stem cell source for mitochondrial transfer and provide a protective mechanism to save damaged cells from mitochondrial dysfunction in response to stress. The transfer of MSC-derived mitochondria to endothelial cells can repress apoptosis after an IR injury by restoring aerobic respiration. The results of studies investigating this transfer are encouraging, paving the way for stem cell transplantation therapy for ischemic stroke [66]. An in vivo study co-cultured hypoxia-induced PC12 cells with MSCs. The treatment reduced apoptosis, swelling of mitochondria, and cristae dissipation. Interestingly, CoCl2 significantly improved the efficacy of the mitochondrial transfer [76].

MSCs achieved efficacy of mitochondrial transplantation via intravenous transplantation into a middle cerebral artery occlusion (MCAO) mouse model. Here, retinal ischemia caused ganglion cells to deteriorate, impairing mitochondrial activity. These findings suggest that MSCs ameliorate mitochondrial dysfunction and ganglion cell death, as was observed 14 days after the stroke [38]. In vitro models produce similar results. Co-cultured retinal pigmented epithelium cells and MSCs displayed improved mitochondrial function and provided neuroprotection against OGD [38]. Additionally, the levels of phosphorylated AKT and BCL-XL increased following injection of mitochondria in the middle cerebral artery animal model. The rats injected with exogenous mitochondria demonstrated improved mitochondrial function and motor performance. Moreover, mitochondrial transfer in the MCAO mice triggered cell-surviving signals following ischemia and decreased energy deficits [66]. Therefore, the ability to restore mitochondrial function through mitochondrial transplantation provides insight into alleviating stroke-induced neuronal death.

The effect of OGD on MSCs derived from the perivascular region, cord lining, and Wharton’s jelly of the human umbilical cord also implicated a key role of mitochondria in these specific MSC tissue sources. The mitochondria in the perivascular region MSCs showed the greatest activity while the MSCs from the cord lining demonstrated the highest survival rate. These findings suggest that hUC-MSCs may be a good source for mitochondrial transplantation for ischemic stroke treatment [77]. Additionally, co-culturing human umbilical vein endothelial (HUME) cells with MSCs improves aerobic respiration and TNT formation in in vitro IR injury models [66].

Human induced pluripotent stem cells (iPSCs) are also the subject of recent studies of mitochondrial transfer. PD model astrocytes derived from human iPSCs spontaneously release healthy mitochondria in damaged neuron cultures. This iPSC-induced mitochondrial transfer significantly ameliorates dopaminergic neuron damage and imparts neuroprotective effects [78]. iPSC’s ability to attenuate neuronal damage through the intercellular transfer of mitochondria warrants further investigation into their therapeutic potential for ischemic stroke.

Spinal cord injury (SCI) models of mitochondrial transfer via bone marrow mesenchymal stem cells (BMSC) show promising results as well. Symptoms of both diseases are very similar: hypoxia, mitochondrial degeneration, oxidative stress, vascular injury, and axonal degeneration [79]. Healthy mitochondria are transferred from BMSCs to motor neurons when these cells are co-cultured in OGD conditions and transplanted into SCI rodents. The application of retinoic acid to this model increases gap junction intercellular communication (GJIC) and the release of mitochondria. In contrast, 18β glycyrrhetinic acid inhibits GJIC and decreases mitochondrial transfer. Mitochondrial transfer from BMSCs coincides with the induction and inhibition of GJIC. After the injured cells successfully integrate the mitochondria, locomotor function, cell survival, and bioenergetics improve [79]. Mitochondrial transfer via BMSCs is effective in treating and improving cardiovascular injury in animals and showcases the potential value of this cell type in stroke therapy. It is possible to transfer viable mitochondria to animal adults and embryonic cardiomyocytes. The transfer of mitochondria upregulates gene expression following an ischemic injury while simultaneously aiding in the reprogramming of adult animal cardiomyocytes. Moreover, treating ischemic human umbilical vein endothelial cells (HUVECs) with BMSC-based mitochondrial transfer therapy limits apoptosis and rescues aerobic respiration [80].

The omnipresent phenomenon of mitochondrial transfer allows for many applications in combination with stem cells. Contemporary literature details the efficacy of mitochondrial transfer in improving stroke, cardiovascular injury, spinal cord injury, and Parkinson’s disease outcomes. The regenerative capacity demonstrated in a variety of tissues warrants its consideration as a primary therapeutic option. In particular, the physiological and pathological improvements seen in treating ischemic stroke illustrate the capability of mitochondrial transfer therapy. However, before moving into clinical trials, the optimal stem cell type must be identified to maximize the efficacy and potency of treatment. Further investigation is needed to discover the true potential of stem cell-based mitochondrial transfer therapy and improve long-term stroke outcomes.

## Figures and Tables

**Table 1:** Recent Discoveries in Mitochondrial Repair for Stroke.

Milestone Discovery	Reference
Measuring mitochondrial respiration	[52,53]
Targeting mitochondria via pharmacological treatment	[54,55,56,57]
Normalizing autophagy	[58,59,60]
Enhancing mitophagy	[61,62,63]
Inhibiting MPTP	[64,65,66,67,68]
Inducing astrocyte-based transfer	[69,70]
Augmenting endogenous tissue repair	[71,72,73,74,75,76,77,78,79,80]

**Table 2:** Overview of Stem Cell-Mediated Mitochondrial Repair for Stroke.

Experimentally Demonstrated Therapeutic Effect	Type of Stem Cell
Transfer of mitochondria via intracellular mechanisms to injured cells	MSC, iPSC, BM-MSC, EPC, ASC
Enhances astrocyte-based transfer	MSC, iPSC, BM-MSC, EPC, ASC
Represses apoptosis after IR injury	MSC
Reverses mitochondrial swelling	MSC
Diminishes mitochondrial cristae dissipation	MSC
Restores ischemic mitochondrial function	MSC
Ameliorates ganglion cell death within 14 days of stroke	MSC
Confers neuroprotection against OGD	MSC
Bolsters TNT formation after IR injury	MSC
Improves mitochondrial survival rate	hUC-MSC
Augments cell bioenergetics and locomotor function	BM-MSC
Rescues aerobic respiration in HUVECs	BM-MSC
Repairs dopaminergic neuron damage	iPSC
